# Carvacrol exhibits rapid bactericidal activity against *Streptococcus pyogenes* through cell membrane damage

**DOI:** 10.1038/s41598-020-79713-0

**Published:** 2021-01-15

**Authors:** Niluni M. Wijesundara, Song F. Lee, Zhenyu Cheng, Ross Davidson, H. P. Vasantha Rupasinghe

**Affiliations:** 1 Department of Plant, Food, and Environmental Sciences, Faculty of Agriculture, Dalhousie University, Truro, NS Canada; 2 Department of Biology, Faculty of Science, Dalhousie University, Halifax, NS Canada; 3 Department of Animal Science, Faculty of Animal Science and Export Agriculture, Uva Wellassa University, Badulla, Sri Lanka; 4 Department of Microbiology & Immunology, Faculty of Medicine, Dalhousie University, Halifax, NS Canada; 5 Department of Applied Oral Sciences, Faculty of Dentistry, Dalhousie University, Halifax, NS Canada; 6 Canadian Center for Vaccinology, Nova Scotia Health Authority, and the Izaak Walton Killam Health Centre, Dalhousie University, Halifax, NS Canada; 7 Department of Pathology, Faculty of Medicine, Dalhousie University, Halifax, NS Canada; 8 Division of Microbiology at the Queen Elizabeth II Health Sciences Centre, Department of Pathology and Laboratory Medicine, Nova Scotia Health Authority, Halifax, NS Canada; 9 National Institute of Fundamental Studies, Kandy, Sri Lanka

**Keywords:** Drug discovery, Microbiology

## Abstract

*Streptococcus pyogenes* is an important human pathogen worldwide. The identification of natural antibacterial phytochemicals has renewed interest due to the current scarcity of antibiotic development. Carvacrol is a monoterpenoid found in herbs. We evaluated carvacrol alone and combined with selected antibiotics against four strains of *S. pyogenes* in vitro. The minimum inhibitory concentration (MIC) and minimum bactericidal concentration (MBC) of carvacrol against *S. pyogenes* were 125 µg/mL (0.53 mM) and 250 µg/mL (1.05 mM), respectively. Kill curve results showed that carvacrol exhibits instantaneous bactericidal activity against *S. pyogenes.* We also demonstrated the potential mechanism of action of carvacrol through compromising the cell membrane integrity. Carvacrol induced membrane integrity changes leading to leakage of cytoplasmic content such as lactate dehydrogenase enzymes and nucleic acids. We further confirmed dose-dependent rupturing of cells and cell deaths using transmission electron microscopy. The chequerboard assay results showed that carvacrol possesses an additive-synergistic effect with clindamycin or penicillin. Carvacrol alone, combined with clindamycin or penicillin, can be used as a safe and efficacious natural health product for managing streptococcal pharyngitis.

## Introduction

*Streptococcus pyogenes,* also known as group A Streptococcus (GAS), a Gram-positive, aerotolerant anaerobic coccus, is a human pathogen responsible for significant morbidity and mortality. *S. pyogenes* is responsible for a myriad of diseases, ranging from mild, non-invasive throat and skin infections^1,2^ to invasive, life-threatening diseases including streptococcal toxic shock syndrome^3,4^ and necrotizing fasciitis^5^. If untreated, *S. pyogenes* infections can develop into severe suppurating infections or non-suppurative complications such as rheumatic heart disease^6–8^. The global prevalence of severe cases is reported over 18 million, with approximately 1.78 million new cases each year^9^. It has been estimated that there are over 500,000 deaths each year globally due to invasive *S. pyogenes* infections^9,10^. *S. pyogenes* has also become one of the top ten infectious causes of mortality by a single organism^11^.

Streptococcal pharyngitis, commonly known as “strep throat,” is responsible for high medical and social costs^12^. Although observed in patients of any age, the prevalence is highest in 5- to 15-year-old children^13^, presumably because of a combination of multiple exposures and low immunity. *S. pyogenes* is responsible for 37% of sore throats in patients < 16 years of age, whereas it is implicated in only 5–15% of adults and 24% of infants less than five years of age^14,15^. Antibiotic therapy is imperative to eradicate *S. pyogenes* from the throat in order to decrease the risk of transmission^15^ and to prevent some of the suppurative and non-suppurative complications^16^. Appropriate antibiotic selection requires consideration of bacteriological and clinical efficacy, the frequency of administration, duration of therapy, potential side effects, patients’ allergies, compliance, and cost^17^. Despite the genetic diversity of *S. pyogenes* and the massive exposure over several decades, the organism remains sensitive to penicillin and other commonly used beta-lactam antibiotics. The Infectious Diseases Society of America, Canadian Pediatric Society, and World Health Organization (WHO) recommend a 10-day course of oral penicillin V (250 mg, 2–3 times/day for children, and 250 mg four times/day or 500 mg twice/day for adults). Beta-lactam antibiotics exhibit bactericidal effects by inhibiting the synthesis of bacterial cell walls^18^. Specifically, they prevent cross-linking between peptidoglycan chains through the DD-transpeptidase enzyme, also known as a penicillin-binding protein^19^.

Although penicillin remains the first choice of drug for *S. pyogenes*, other antibiotics are shown to be effective in eradicating *S. pyogenes*. First-generation oral cephalosporins and macrolides are recommended for patients with penicillin allergy as alternative treatment options^18^. Macrolides bind to 23S ribosomal RNA target sites of *S. pyogenes*, thereby inhibiting protein synthesis. Macrolide resistance has been well described either due to active efflux (*mef* genes) or target modification (*erm* genes)^20,21^.

**Figure 1:**
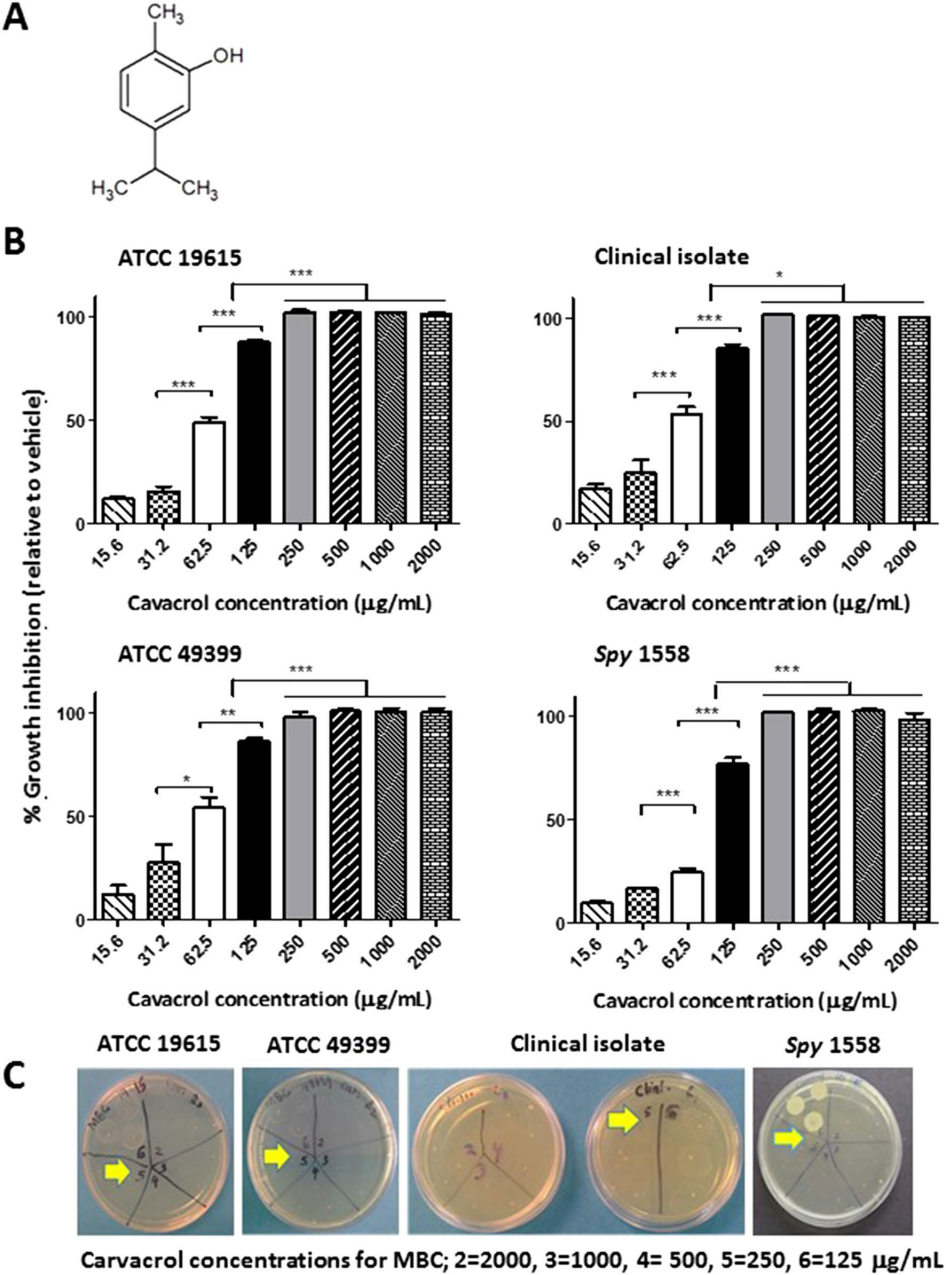
Carvacrol inhibits the growth of planktonic *Streptococcus pyogenes* in a concentration-dependent manner. (**A**) The chemical structure of carvacrol. (**B**) Inhibition of growth of *S. pyogenes* strains (ATCC 19615, ATCC 49399, a clinical isolate from a pharyngeal patient and an erythromycin-resistant *Spy* 1558) by carvacrol at the given concentrations was measured using micro-broth dilution assay in BHI broth after 24 h of incubation at 37 °C. Bacterial turbidity was measured at OD = 600 nm, and percentage growth inhibition is expressed relevant to the vehicle (0.25% DMSO) as the mean ± SE of three independent experiments. Statistical analysis was performed using one-way ANOVA, and the differences among means were compared using Tukey’s test; *P < 0.05, **P < 0.01, and ***P < 0.001. (**C**) Bactericidal concentrations of carvacrol for *S. pyogenes* strains were determined by inoculating on agar plate from each replicates well in broth dilution plate that shows a complete absence of growth and was incubated at 37 °C for 24 h. Concentrations labeled as 2, 3, 4, 5, and 6 on the agar plate represent carvacrol concentration of 2000, 1000, 500, 250, and 125 µg/mL, respectively. Arrows indicate the MBCs observed. MBC, Minimum bactericidal concentration.

Carvacrol, also known as cymophenol (2-methyl-5-propan-2-ylphenol, Fig. 1A), is a monoterpene phenolic compound of *Thymus* and *Oregano* family of herbal plants^22–24^. Carvacrol or carvacrol containing essential oils have been extensively studied for biological activities such as anti-oxidant^25^, anti-inflammatory^26^, anti-cancer^25^, anti-pyretic^27^, and analgesic properties^27^. Carvacrol exhibits antimicrobial activities against yeast/fungi^24,28^, Gram-positive^29^, and mostly Gram-negative bacteria^23,30^. In Gram-negative bacteria, carvacrol depolarizes the cytoplasmic membranes^31,32^. Furthermore, carvacrol appears to affect ATP synthesis and subsequently reduce the other energy-dependent cellular processes such as the synthesis of enzymes and toxins^33^. However, the antibacterial mechanisms of carvacrol against human pathogenic Gram-positive bacteria have not been reported sufficiently.

Low toxicity and low cost of production of carvacrol make it an attractive food additive. European Union Food Improvement Agents and Joint FAO/WHO Expert Committee on Food Additives (JECFA), have classified carvacrol as a flavoring agent^34,35^. The specific objectives of the study were to investigate the efficacy of carvacrol against *S. pyogenes,* understand the mechanism of action on cell integrity, determine cytotoxicity to human cells, and assess the combined effect with four antibiotics.

## Results

### Carvacrol inhibits the growth of *S. pyogenes*

The antibacterial activities of carvacrol and four antibiotics against *S. pyogenes* are summarized in Table 1. Carvacrol showed growth inhibitory effects against all four tested strains of *S. pyogenes* with a minimum inhibitory concentration (MIC) of 125 μg/mL. The percentage of growth inhibition relative to dimethyl sulfoxide (DMSO, vehicle) control is shown in Fig. 1B. Both micro-and macro-broth dilution methods revealed similar findings.

**Table 1:** Minimum inhibitory concentration (MIC) and minimum bactericidal concentration (MBC) (μg/mL) of carvacrol against four strains of *Streptococcus pyogenes*.

Drug	Strains of *Streptococcus pyogenes*							
	ATCC 19615		ATCC 49399		Clinical isolate		*Spy* 1558	
	MIC^a^	MBC^b^	MIC^a^	MBC^b^	MIC^a^	MBC^b^	MIC^a^	MBC^b^
Carvacrol	125	250	125	250	125	250	125	250
Penicillin G	0.008	0.016	0.008	0.016	0.008	0.016	0.008	0.016
Penicillin Vk	0.008	0.016	0.008	0.016	0.008	0.016	0.008	0.016
Clindamycin	0.031	0.063	0.031	0.063	0.031	0.063	0.250	0.500
Erythromycin	0.125	0.250	0.125	0.250	0.125	0.250	15.63	31.25

Data are representative of three independent experiments.

^a^Minimum inhibitory concentration (MIC) was determined as the lowest concentration of tested compound that inhibited bacterial growth.

^b^Minimum bactericidal concentration (MBC) was determined as the lowest concentration of tested compound that killed at least 99.9% of the initial inoculums.

### The activity of carvacrol with conventional antibiotics and among antibiotic combinations

The antibacterial activity of carvacrol in combinations with antibiotics was analyzed by the checkerboard assay (Fig. S1). The fractional inhibitory concentration index (FICI) values for different combinations of carvacrol, penicillin G salt, penicillin Vk, clindamycin, and erythromycin are summarized in Table 2. Carvacrol displayed marginal additive synergism with clindamycin, with a FICI value of 1.0 against *S. pyogenes*, whereas the combinations with other antibiotics showed no significant synergistic effects.

**Table 2:** The fractional inhibitory concentration (FIC) and FIC index (FICI) for the carvacrol and antibiotic combinations against *Streptococcus pyogenes*.

Combinations		ATCC 19615				Clinical isolate			
A	B	FIC A	FIC B	ΣFICI	Interpretation	FIC A	FIC B	ΣFICI	Interpretation
Carvacrol	Penicillin G	0.5	1	1.5	IND	0.5	1	1.5	IND
Carvacrol	Penicillin Vk	0.5	1	1.5	IND	0.5	0.5	1	ADD
Carvacrol	Clindamycin	0.5	0.5	1	ADD	0	1	1	ADD
Carvacrol	Erythromycin	0.25	1	1.25	IND	–	–	–	–

Fractional inhibitory concentrations (FICs) and fractional inhibitory concentration index (FICIs) were calculated FICI = ΣFIC = FIC_A_ + FIC_B_ where, MIC_A_ value is the MIC of compound A alone, MIC_B_ value is the MIC of compound B alone, and MIC_A+B_ value is the MIC of compound A in the presence of compound B, and vice versa for MIC_B+A_.

FICIs were interpreted as SYN, synergy (FICI ≤ 0.5); ADD, additive synergy (> 0.5 FICI ≤ 1.0); IND, indifference/no interaction (> 1.0 FICI ≥ 4.0); and ANT, antagonism (FICI Index > 4.0).

### Carvacrol instantaneously kills *S. pyogenes*

**Figure 2:**
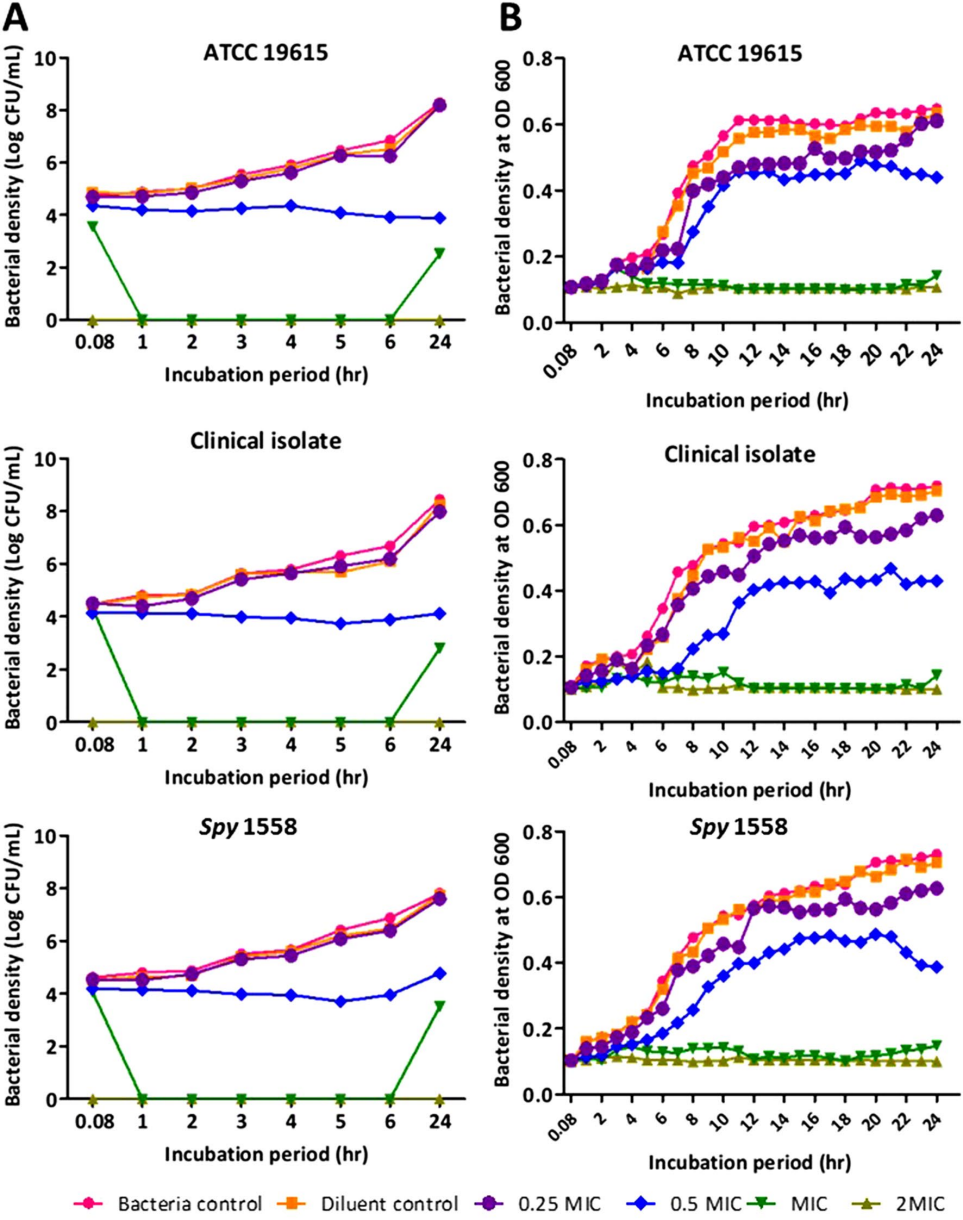
Carvacrol exerts concentration-dependent growth inhibition on *Streptococcus pyogenes* and an instantaneous bactericidal effect at 2 × MIC. Time-kill curve plots from (**A**) agar plate count method* and (**B**) spectrophotometric method** on the growth of *Streptococcus pyogenes* ATCC 19615, clinical isolate, and erythromycin-resistant, *Spy* 1558 in the presence of ¼ × MIC ½ × MIC, 1 × MIC, and 2 × MIC of carvacrol or 0.25% DMSO (diluent control) and no treatment (bacteria control). The bacteria and carvacrol solutions were prepared in BHI broth and were assessed for a period of 24 h at a 37 °C incubation period. *Cell growth/killing at 0.08, 1, 2, 3, 4, 5, 6, and 24 h incubation was measured by performing viable cell counts by dilution of cultures in saline solution (0.85% NaCl) and enumeration on BHI agar plates in duplicate. **The bacterial turbidity at OD = 600 nm was measured using a spectrophotometer in 1 h intervals over 24 h incubation period with different carvacrol concentrations or diluent in triplicate. Both methods were performed in three independent experiments. “0” in the scale represents “below the limit of detection”.

The bactericidal effect of carvacrol on the planktonic growth of *S. pyogenes* (ATCC 19615, clinical isolate, and *Spy* 1558) was investigated, and the minimum bactericidal concentration (MBC) values are shown in Table 1 and Fig. 1C. The effect of carvacrol against *S. pyogenes* was investigated by studying time-kill kinetics (Fig. 2), and carvacrol showed concentration- and time-dependent bacterial killing ability. The complete killing of *S. pyogenes* was observed immediately after exposure to the carvacrol at the concentration of 250 μg/mL (2 MIC = MBC), and such killing was observed for all three strains. A potent killing was observed with 125 μg/mL (MIC) of carvacrol throughout the incubation; however, complete bacterial killing was not persisted until 24 h. Furthermore, a significant growth inhibition activity was observed at 1/2 × MIC of carvacrol. However, reducing the bacterial count at the end of the 24-h incubation period did not go beyond 3 to 4 *log* units for three strains. Both methods (viable cell counts method (Fig. 2A) and spectrophotometric method (Fig. 2B)) gave similar results.

### Carvacrol induces morphological changes in *S. pyogenes*

**Figure 3:**
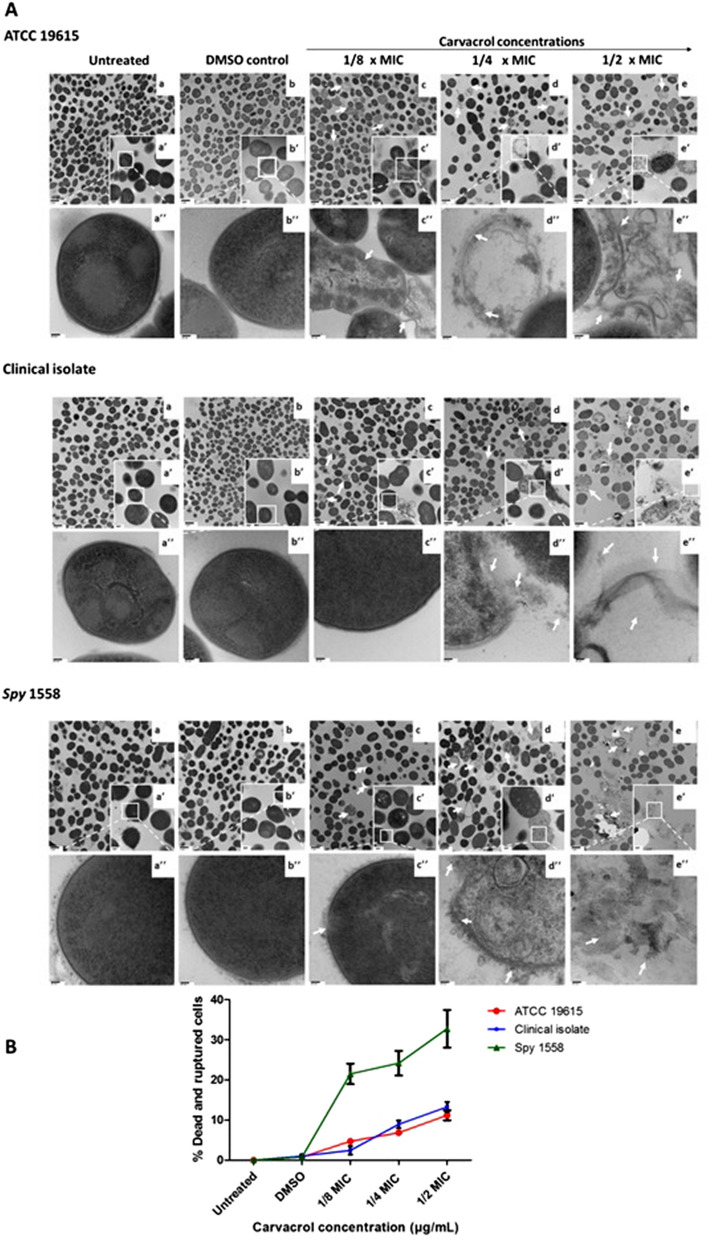
Transmission electron microscopic micrographs of three strains of *Streptococcus pyogenes* treated with or without exposure to carvacrol. (**A**) Cell morphology of vehicle control (DMSO) (b,b′,b′′), carvacrol at 1/8 × MIC (c,c′,c′′), carvacrol at ¼ × MIC (d,d′,d′′), and carvacrol at ½ × MIC (e,e′,e′′) were compared with untreated bacterial (a,a′,a′′) in BHI media. The exponential phase of *S. pyogenes Spy* 1558 cells was treated with different concentrations of carvacrol for 16 h. Images were taken at magnifications of × 10,000, × 50,000 and × 100,000 for a,b,c,d,e , a′,b′,c′,d′,e′’ and a′′,b′′,c′′,d′′,e′′, respectively. Arrows indicate dead cell morphologies. *MIC* minimum inhibitory concentration, *BHI* brain heart infusion. (**B**) Percentage of carvacrol-induced morphological damages determined by TEM. An average of 200 cells from two independent experiments were analyzed per each treatment. Morphological changes were quantified as the percentage of ruptured and dead cells to the total cells.

Transmission Electron Microscopy (TEM) was employed to observe the morphological and ultrastructural alterations induced in three *S. pyogenes* strains upon exposure to 1/8 × MIC, ¼ × MIC, and ½ × MIC of carvacrol treatment compared to the control. Both untreated control (Fig. 3Aa,a′,a′′) and vehicle control (Fig. 3Ab,b′,b′′) cells remained intact with a complete cell wall, a visible cell membrane, and a homogeneous cytoplasm. In contrast, significant cell deaths (indicated in white arrows) such as ruptured or completely broken cell wall, detached cytoplasmic membrane from the cell wall, dispersion of the intracellular contents, and noticeable cytoplasmic clear zones were observed in ½ × MIC-carvacrol treated ATCC 19615, clinical isolate and *Spy* 1558 (Fig. 3Ac,c′,c′′) cells. The percentage of ruptured and dead cells relative to the total cells was calculated using images of TEM (Fig. 3B). Cells with the distorted shape compared to the smooth spherical shape of control were also observed. Cellular debris and broken cell wall parts were also abundant.

Morphologies of dead and disintegrated cells including, membrane fusing, clumping, ruptured, the disintegration of the cell wall and/or membrane, cytoplasmic disruptions of cells following exposure to ¼ × MIC carvacrol were noticeable in *S. pyogenes* strains (Fig. 3Ad,d′,d′′). The round shape of most of the intact cells turned into abnormally elongated shapes with ruptured or broken cell walls. The ruptured and dead cell-morphologies were observed in all three bacterial strains; however, % ruptured and dead cells was significantly higher in *Spy* 1558 than in other strains (Fig. 3B). Carvacrol at or greater than MIC was highly bacteriocidal; therefore, it did not generate enough bacterial cell pellets for TEM analysis. TEM images also confirmed that cell density was significantly reduced in all treated samples compared to vehicle control in a concentration-dependent manner.

### Carvacrol induces cytoplasmic content leakage

The membrane leakage assays were carried out to assess the integrity of the cell membranes due to cytoplasmic membrane damage of *S. pyogenes*. The release of RNA, double and single-strand DNA was detected only in the supernatants of 125 μg/mL (MIC) of carvacrol treatment. The concentrations of RNA, dsDNA, and ssDNA released by ATCC 19615 were estimated to be 87 ± 0.0 ng/µL, 156 ± 19.3 ng/µL, and 82 ± 3.7 ng/µL, respectively, while that by the clinical isolate were 85 ± 2.4 ng/µL, 71 ± 8.2 ng/µL, and 69 ± 2.5 ng/µL, respectively.

**Figure 4:**
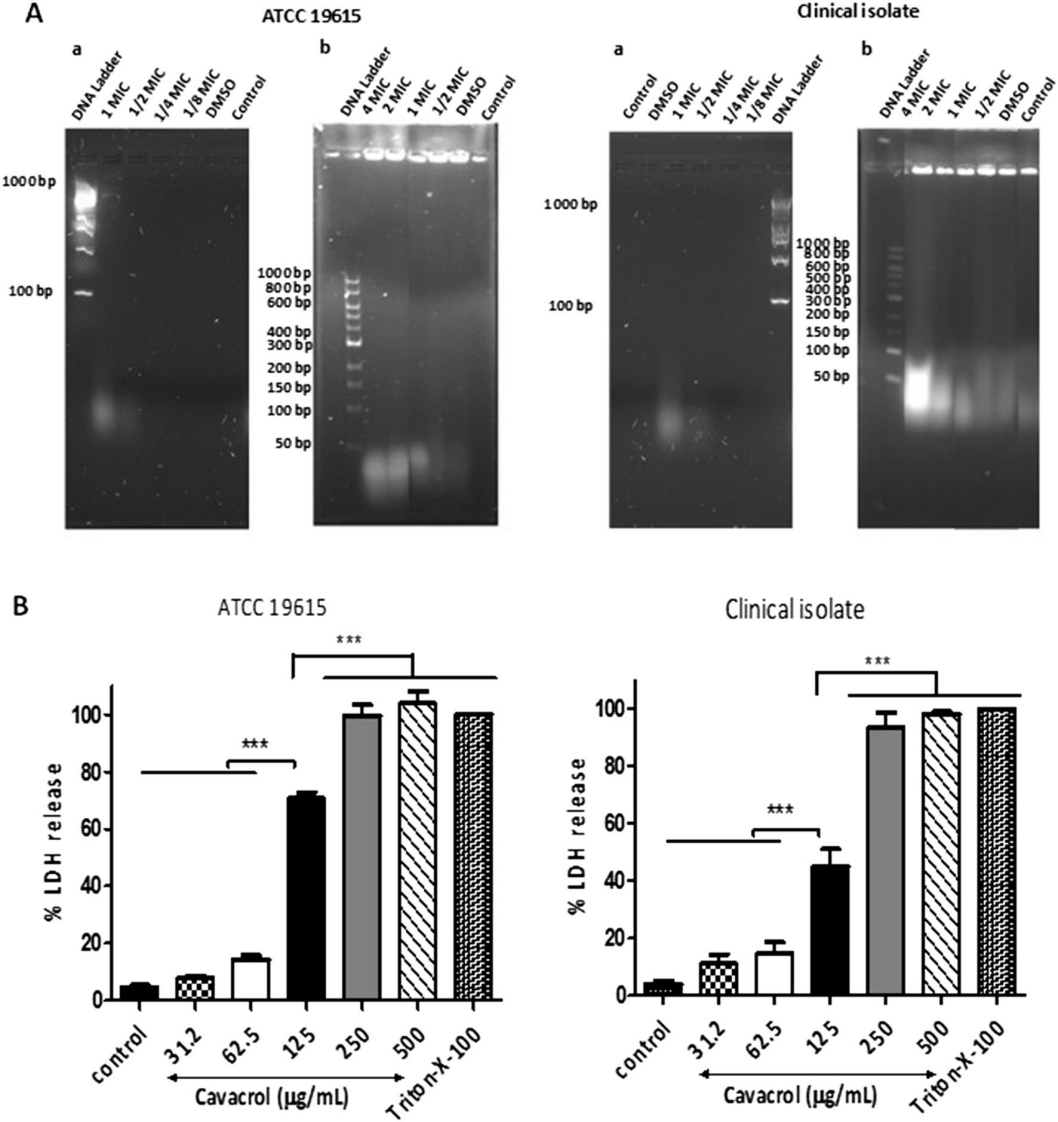
Carvacrol induces cytoplasmic content leakage. (**A**) Carvacrol causes the leakage of cytoplasmic nucleic materials from *Streptococcus pyogenes* in a concentration-dependent manner. Agarose gel (1%, w/v) electrophoresis and gel red staining of leaked nucleic acid from cell suspensions (1 × 10^6^, OD = 0.02) of ATCC 19615 and clinical isolate strains (a) exposed to MIC (125 μg/mL), 1/2 MIC, and 1/4 MIC of carvacrol or vehicle control (0.25% DMSO) over 24 h. 1 kb ladder as reference. (b) Agarose gel (0.8%, w/v) electrophoresis and ethidium bromide staining of genomic DNA recovery of the DNA by ethanol precipitation from bacteria suspension (OD = 0.6) of followed by 2 h carvacrol treatment. Carvacrol concentration is adjusted to the high bacterial density as MIC = 3750 μg/mL and 1 kb ladder were used as a reference. (**B**) Carvacrol causes leakage of lactate dehydrogenase (LDH) from ATCC 19615 and a clinical isolate of *Streptococcus pyogenes. *Overnight incubated cells were treated with different carvacrol or vehicle (0.25% DMSO) for 4 h. A standard lysis buffer (9% Triton X-100) was included as a positive control and was defined as 100% LDH release. The carvacrol-induced LDH release into culture media was measured using the Promega LDH Cytotoxicity Assay Kit. Data expressed as % LDH release and represented mean ± SE (n = 3), ***P < 0.001, compared among means (ANOVA, Tukey’s test). (**C**) Carvacrol does not cause the release of LDH from cultured human tonsil epithelial cells. The seeded human tonsil epithelium cells (TonEpiCs) for 24 h were treated with different concentrations of carvacrol or DMSO vehicle for 4 h. A standard lysis buffer was included as a positive control and defined as 100% LDH release. The carvacrol-induced LDH release into cell supernatant was measured using the Promega LDH Cytotoxicity Assay Kit. Data expressed as % LDH release and represented mean ± SEM (n = 3), ***P < 0.001, *P < 0.05, compared among means (ANOVA, Tukey’s test).

Since some 260–280 nm absorbing proteins in cell supernatants can interrupt the absorbance measurement of nucleic acids, to confirm the carvacrol-induced cytoplasmic nucleic acid release, ethanol precipitation was carried out and then was visualized, followed by agarose gel electrophoresis. When ATCC 19615 and the clinical isolate were grown in the presence of 62.5 μg/mL and 125 μg/mL concentrations (1/2 × MIC and MIC, respectively) of carvacrol, cells released visualizable nucleic acids after 24 h (Fig. 4Aa). When high-density bacterial pellets were treated with carvacrol for 1 h, the concentration-dependent release of nucleic acid was observed (Fig. 4Ab).

Leakage of a common cytosolic enzyme, L-lactate dehydrogenase (LDH), into the cell medium was measured. The LDH release by *S. pyogenes* treated with 125 μg/mL of carvacrol was estimated to be 47–71% relative to the total cellular LDH, which was significantly higher than the vehicle control (Fig. 4B). Furthermore, the amount of LDH released by carvacrol concentration of 250 μg/mL or more was not significantly different with LDH released by Triton X-100 treated. These results suggest the concentration-dependent disturbance to membrane integrity compromise by carvacrol. In contrast, carvacrol did not cause the release of LDH from cultured human tonsil epithelial cells (Fig. 4C).

### In vitro cell cytotoxicity of carvacrol

**Figure 5:**
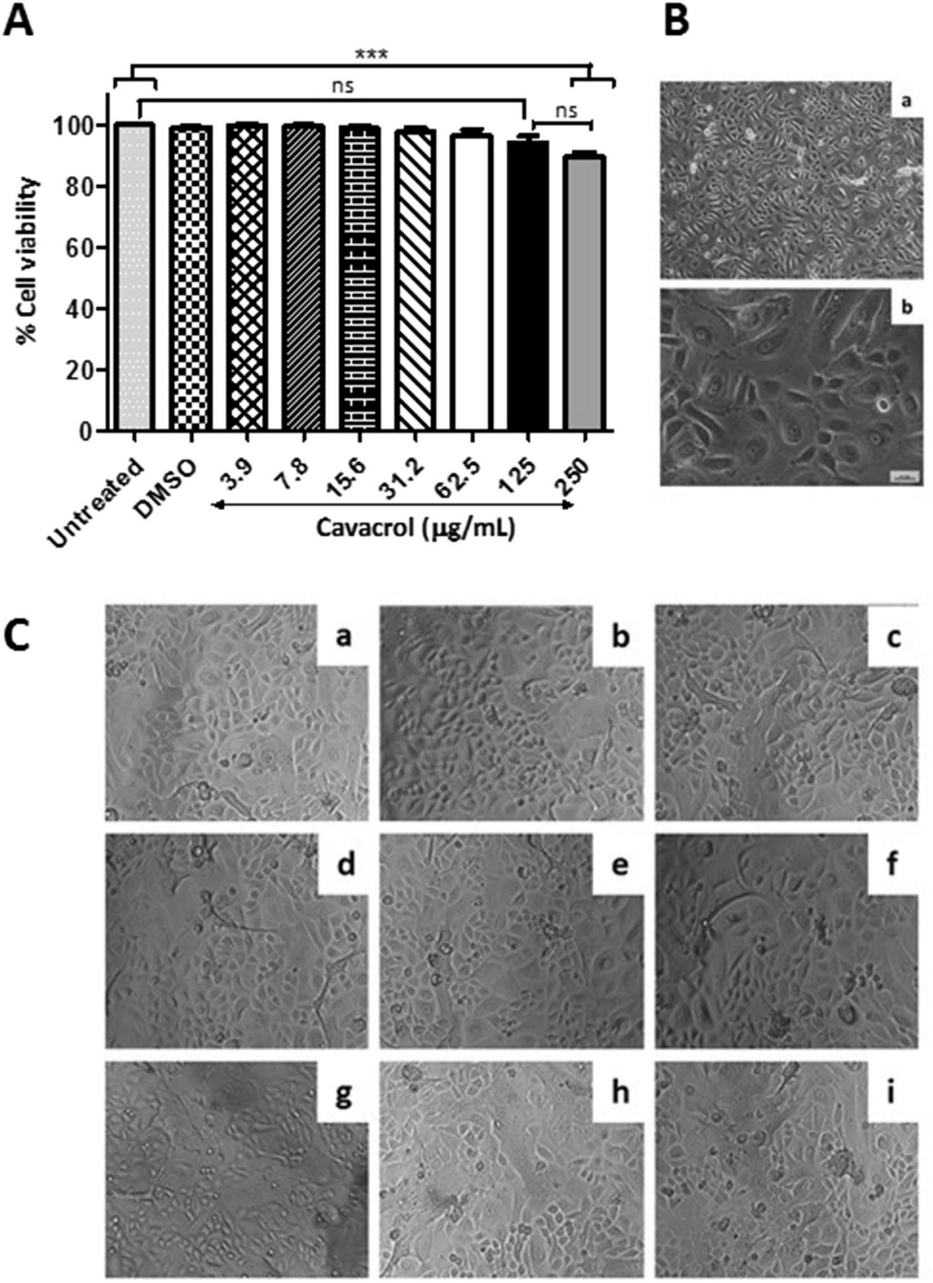
Carvacrol is not cytotoxic to the human tonsil epithelium cells. (**A**) Cell viability was measured using the MTS assay after treating human tonsil epithelium cells (TonEpiCs) with 15.6, 31.3, 62.5, 125, and 250 µg/mL carvacrol for 24 h. Absorbance was measured at 490 nm. Data are shown as mean ± SE from three independent experiments, each in triplicate. Differences among means were compared with untreated TonEpiCs (ANOVA, Tukey’s test). (**B**) Morphology of TonEpiCs grown in poly-l-Lysine coated flask with tonsil epithelial growth medium at 90% confluence was observed using a phase-contrast microscope. (a) Magnification, × 100; (b) Magnification, × 400. (**C**) TonEpiCs treated with different carvacrol were photographed using a phase-contrast microscope at × 100 magnification. Representative photographs captured at 24 h post-treatment in three independent experiments are shown. (a) untreated; (b) vehicle control (0.25% DMSO) and carvacrol treatments of (c) 3.9 µg/mL, (d) 7.8 µg/mL, (e) 15.6 µg/mL, (f) 31.3 µg/mL, (g) 62.5 µg/mL, (h) 125 µg/mL, and (i) 250 µg/mL.

The results showed that carvacrol was not cytotoxic for normal human tonsil epithelium (HTonEpiCs) cells at concentrations below or equal to 250 μg/mL (Fig. 5A). The viability of HTonEpiCs cells was determined to be 89%. The rest of the carvacrol concentrations tested exhibited safety, as > 95% of the cells were viable following carvacrol treatments (Fig. 5). The cell morphology agreed with the above observations (Fig. 5B,C). Results demonstrated that carvacrol within the tested range of concentration is not toxic to mammalian cells (Fig. 4C) but rather specific toward bacterial cells.

## Discussion

For thousands of years, natural compounds have been used to treat infectious diseases worldwide^36,37^. Carvacrol is a monoterpenoid phenolic derivative primarily found in essential oils of herbal plants, including thyme and oregano. Extensive studies have been conducted investigating the biological properties of carvacrol for its potential use in clinical applications^23,25,26^. In this study, we investigated the antimicrobial activity of carvacrol against *S. pyogenes* and attempted to understand the potential mechanisms of action. The MIC and MBC values (125 and 250 μg/mL, respectively) for four strains coincide with the results of a previous study^38^ that reported MICs of carvacrol ranged from 64 to 256 μg/mL against clinical isolates of *S. pyogenes* isolated from children with pharyngotonsillitis in Italy. Our previous study with oregano essential oils that consist of more than 90% of carvacrol exhibited a similar bactericidal activity^22^. Similar to many other essential oil compounds, carvacrol's antimicrobial activity is related to its phenol structure and the presence of delocalized electrons^39^.

The synergistic antibacterial activity of various plant extracts and phytochemicals in the presence of conventional antibiotics has been reported. The mechanism of synergistic actions of plant extracts and phytochemicals is postulated as a modification of active sites on bacterial cells, inhibition of enzymes that catalyze modification or degradation of antibiotics, inhibition of efflux pumps, or increase of membrane permeability^40–42^. The ability of essential oils containing carvacrol, cinnamaldehyde, cinnamic acid, eugenol, and thymol to exert synergistic effects combined with several antibiotics, including macrolides, has also been reported^43^. However, in contrast to the evidence of synergism between several different combinations of erythromycin and carvacrol reported in Magi et al.^38^ we have not observed a synergistic effect of tested erythromycin and carvacrol combinations over all the strains. However, among all the combinations, only additive synergy, were seen in some concentrations of carvacrol and penicillin Vk as well as carvacrol and clindamycin. The effective carvacrol-antibiotics combinations of the current study can be further investigated using killing time, cell targets, and mechanisms of action, considering them as a potential therapeutic strategy for common drug-resistant bacteria.

The time-kill assay is a robust and appropriate tool for collecting information about the dynamic interaction between the antibacterial agent and the bacterial strain. Therefore, bacterial killing kinetics of a range of carvacrol concentrations at different times were assessed. Carvacrol-treated *S. pyogenes* was killed within 5 min exposure at concentrations over 250 μg/mL (1.05 mM), whereas low concentrations (≤ 125 μg/mL) of carvacrol inhibits the growth gradually via a sublethal phase. Magi et al.^38^ reported that several dead cells were detected as early as 1 h after incubation with carvacrol at the MIC using live/dead assay. We had previously investigated that time taken to accomplish complete *S. pyogenes* killing by penicillin G. When the MIC concentration of penicillin G (2 × MIC = MBC = 0.016 μg/mL) was used in the kill curve against *S. pyogenes*, 24 h were taken for the complete bacterial killing^22^. The observed instantaneous bactericidal action of carvacrol in this study suggests that carvacrol may have affected the bacterial membrane integrity. On the other hand, the observed time-dependent killing and growth inhibition by carvacrol indicate that additional mechanisms can be involved in cellular processes, such as inhibition of protein, nucleic acids, and lipid synthesis.

Ultrastructural morphological abnormalities induced by carvacrol were observed by electron microscopy. Comparisons of TEM images over their respective untreated controls revealed that both cell wall and membrane destruction by carvacrol and leakage of bacterial cytoplasmic contents when bacterial cells were exposed to carvacrol. The changes were evident with an increase in the concentration, which was consistent with the time-kill study results and cell leakage assays. These observations are parallel to the scanning electron microscopy observations of a previous study with carvacrol and thymol, an isomer of carvacrol, which was also able to disturb the *S. mutans* membrane and caused the release of cellular contents^44^. Moreover, a similar membrane destructive activity of carvacrol against another Gram-positive bacteria, *Staphylococcus aureus* ATCC 43300, was reported previously^45^. The scanning electron micrograph-observations showed that carvacrol treated cells became rough and wrinkled with depression appearing on their surfaces at the concentration of 1.03, 2.06, and 4.12 mM for 4-h treatment^45^. However, our TEM observation shows more detailed sectional views of the interior of an intact and damaged cell structure instead of the cell surface changes.

Leakage of nucleic acids and LDH enzymes are indicators that confirm the effect of carvacrol on cytoplasmic leakage of *S. pyogenes* cells. Carvacrol caused complete or severe damage to the membrane at higher concentrations and increased the leaching out of cytosolic proteins, enzymes, nutrients, and genetic materials. Therefore, correlations between leakage‐inducing concentrations of carvacrol and MIC value suggest that membrane damage is an important mechanism of action. Similarly, monoterpenes cooperate with the lipid bilayer of cytoplasmic membranes affecting membrane leakage of cellular contents such as ATP, ions, and nucleic acid^45–47^. Wang et al.^45^ discussed the carvacrol-induced intracellular components leakage using β-galactosidase as an indicator. These observations showed that carvacrol treatment affected the integrity of *S. aureus* cell membranes, which likely resulted in a decrease in cell viability. Their findings were in line with our present results of concentration-dependent lactate dehydrogenase leakage in carvacrol treated *S. pyogenes* cells. Therefore, we could suggest that carvacrol's cell membrane damage mechanism would not differ between these two species. Besides increasing the permeability of the bacterial cell membrane, it was suggested that carvacrol directly binds to genomic DNA as the second key mechanism of action^45^. Future studies need to be aimed at understanding DNA damage in *S. pyogenes* cells by carvacrol. The ability of carvacrol to cause leakage of cellular contents suggests that its action may cause pores in the bacterial membranes. It is widely believed that the antimicrobial action of many small molecules results in the formation of pores in the bacterial membranes and cause leakage of cellular contents^48^.

Carvacrol has also been reported to be safe and exert minimal toxicity on human cells^49^. We confirmed that carvacrol exhibits high selective cytotoxicity towards bacterial cells over human tonsil epithelial cells. This bacterial selectivity might be attributed to different physicochemical properties of membrane components, especially phospholipids found in bacteria versus mammalian cells. Cholesterol is the major lipid component of the eukaryotic membrane but not of the bacterial cell membrane and may lead to discrimination between bacterial and host cell membranes^50^.

As in all Gram-positive bacteria, the cell wall of *S. pyogenes* is composed of thick peptidoglycan (PGN) covered with proteins, teichoic acid, and lipoteichoic acid (LTA)^51,52^. Hence, carvacrol must cross the bacterial cell wall before interacting with the cytoplasmic membrane; however, the role of the cell wall in interacting with carvacrol to be revealed. We can postulate that cell wall PGN and teichoic acid may allow penetration of carvacrol to the cytoplasmic membrane. Therefore, future studies can be targeted to understand the effect of carvacrol on inhibition of PGN biosynthesis and induction of PGN degradation. The LTA present in most Gram-positive bacterial species, including *Streptococcus*^53^. Fatty acid chains in the LTA are anchored in the membrane, whereas the remaining part of LTA (glycerol phosphate or ribitol-phosphate chain) hangs out through the cell wall. Lipophilic ends of these LTA are found on the surface of the cell wall might facilitate the easy penetration of small monoterpene hydrophobic compounds such as carvacrol^54^ to the cell membrane, and accumulation of carvacrol in the membrane is expected. Therefore, these accumulated higher carvacrol concentrations explain the membrane disruption and instant bactericidal effect. Bacteria would certainly need either modification of the charge of cytoplasmic membrane lipids or its composition to become resistant to those membrane-active antibacterial agents^55^. However, our results indicated that bacteria are susceptible to carvacrol. Carvacrol exerts its bactericidal and inhibitory activities against *S. pyogenes* cells by disturbing cytoplasmic membrane integrity. Due to the potential bacterial membrane-targeted biocidal action, carvacrol can be assessed as a novel approach to treat drug-resistant pathogens such as erythromycin-resistant *S. pyogenes*.

Our results show that the efficacy of carvacrol on disrupting the cell membrane integrity of *S. pyogenes*. However, to validate potential carvacrol applications as a new antibacterial agent, other potential mechanisms such as inhibition of macromolecular synthesis of *S. pyogenes*, inhibition of biofilm formation, and inhibition of quorum sensing, and preventing adhesion needs to be further investigated. The effects of carvacrol on bacterial membrane proteins, nucleic acid, and enzymes that are involved in membrane lipid biosynthesis and degradation need to be explored. Based on the observations, we suggest carvacrol as a potential antibacterial agent to treat or manage infections caused by *S. pyogenes*. Since carvacrol is listed as a Generally Recognized as Safe (GRAS) food additive by the United States Food and Drug Administration^56^, a significant potential exists for carvacrol as a safe molecule for broader therapeutic applications.

## Conclusion

This study investigated the antibacterial effect of carvacrol against *S. pyogenes*, particularly potential growth inhibition and rapid bactericidal mechanisms. Carvacrol at 250 μg/mL (1.05 mM) exhibited instantaneous bactericidal activity against three tested strains of *S. pyogenes*. Our study revealed that carvacrol kills *S. pyogenes* primarily by compromising the cell membrane integrity, leading to cytoplasmic content leakage and, ultimately, bacterial cell death. These findings suggest that carvacrol has the potential to develop as a novel natural health product in the forms of throat vapor, lozenge, or mouthwash to manage the discomfort associated with streptococcal pharyngitis. Furthermore, carvacrol can be further explored as a promising antibacterial agent with higher cell selectivity for potential clinical applications against drug-resistant pathogens.

## Material and methods

### Media, chemicals, and bacterial strains

Carvacrol, penicillin G sodium salt, DMSO (≥ 99.8%), LTA, ethidium bromide (3,8-diamino-5-ethyl-6-phenylphenanthridinium bromide, > 95%), poly-l-lysine (PLL), [3-(4,5-dimethylthiazol-2-yl)-5-(3-carboxymethoxyphenyl)-2-(4-sulfophenyl)-2H-tetrazolium] (MTS) and phenazine methosulfate (PMS) were obtained from Sigma-Aldrich Ltd., Oakville, ON, Canada and peptidoglycan (PGN) were from Cedarlane Laboratories, Burlington, ON, Canada. Four *S. pyogenes* strains ATCC 19615, and ATCC 49399, a clinical isolate (originated from a pharyngitis patient) and an erythromycin-resistant strain (*Spy* 1558, erm) were used in the study and were grown in brain heart infusion (BHI), Oxoid Ltd., Nepean, ON, Canada. Cultures storage, sub-culturing, and inoculum preparation (1 × 10^6^ CFU/mL) were performed as described previously^22^.

### Growth inhibition assays

#### Macro-dilution method

The MIC was determined according to the method of the Clinical and Laboratory Standards Institute (CLSI). Two-fold serial dilutions of carvacrol (0.125 to 2000 μg/mL) were made from stock in DMSO and combined with *S. pyogenes* suspension. The tube containing bacteria in 1% DMSO served as controls. The MIC was the lowest carvacrol concentration with no visible growth in tubes after 24 h incubation at 37 °C.

#### Micro-dilution method

The micro-broth dilution assay was conducted the same as the above method but in 96-well plates as per the guidelines of CLSI. Carvacrol (0.125 to 2000 µg/mL) was incubated along with bacteria in 200 µL of total volume. Absorbance was measured at OD_600_ after 24 h incubation at 37 °C.

### Bactericidal activity

The MBC was determined by pipetting 30 μL from wells that showed no visual growth in MIC experiments onto BHI agar, and the plates were incubated at 37 °C for 24 h. The lowest carvacrol concentration with no visible bacterial colonies (assumed to eliminate ≥ 99.9% of the initial inoculum) was considered the MBC.

### Synergistic effect of carvacrol

The synergistic effects of carvacrol with antibiotics were assessed by the checkerboard method^57^. Briefly, 50 μL of each antibiotic and carvacrol (from twofold serial dilutions) were added into 100 μL of bacterial suspension in a 96-well plate. OD_600_ was measured after 24 h incubation. Fractional Inhibitory Concentration Index (FICI) was calculated according to the formula: FICI_A_ = [(MIC A Combination)/ MIC A along]

$${\text{FICI}}_{{\text{A}}} = {\text{ MIC}}_{{{\text{A}} + {\text{B}}}} /{\text{MIC}}_{{\text{A}}} ,{\text{ FIC}}_{{\text{B}}} = {\text{ MIC}}_{{{\text{B}} + {\text{A}}}} /{\text{MIC}}_{{\text{B}}} ,$$

$${\text{FICI }} = {\text{S}}\left[ {\left( {{\text{MIC}}_{{\text{A}}} \;{\text{combination}}} \right)/{\text{ MIC A along}}} \right] \, + \, \left( {{\text{MIC}}_{{\text{B}}} \;{\text{combination}}} \right)/{\text{ MIC B }}\;{\text{along}}$$

$${\text{FICI }} = {\text{ FICI}}_{{\text{A}}} + {\text{FICI}}_{{\text{B}}}$$

where, MIC_A_ value is the MIC of compound A along, MIC_B_ value is the MIC of compound B alone, and MIC_A+B_ value is the MIC of compound A in the presence of compound B, and vice versa for MIC_B+A_. FICI values were interpreted accordingly as synergy (FICI ≤ 0.5), additive synergy (> 0.5 FICI ≥ 1.0), Indifference /No interaction (> 1.0 FICI ≥ 4.0), and antagonism (FICI > 4.0).

### Time-kill analysis

#### Spectrophotometric method

The bactericidal effect of carvacrol on the planktonic growth of *S. pyogenes* (ATCC 19615, clinical isolate, and *Spy* 1558) was determined by time-kill curve analyses as previously described^22^. *S. pyogenes* were grown with (1/4 × MIC, 1/2 × MIC, MIC, and 2 × MIC) or without carvacrol in 100 μL of BHI in 96-well plates. The bacterial suspension was introduced and was incubated at 37 °C. Growth dynamics were measured spectrophotometrically (at optical density = 600 nm) every hour for 24 h.

#### Viable cell counts method

*S. pyogenes *(ATCC 19615, clinical isolate, and *Spy* 1558) cell suspensions (100 µL) were incubated in the presence of carvacrol or penicillin G in 96-well plates. Bacteria in DMSO (vehicle) and BHI (untreated) served as controls. The plates were incubated at 37 °C in a humid 5% CO_2_-enriched atmosphere with shaking. Bacterial growth was monitored over a 6 h period. At selected time points, the viable bacterial count was measured using a previously described method^58^ with some modifications. Bacterial suspensions were diluted in sterile saline water (1:9) and 20 μL drop-spotted onto BHI agar. Colonies were counted after 24 h-incubation. The time taken to kill initial bacterial loads were assessed by plotting the *log* CFU/mL versus incubation time.

### Transmission electron microscopy (TEM)

The morphological changes of *S. pyogenes* were observed following incubation with carvacrol (1/4 × MIC, 1/2 × MIC, and MIC). After 16 h, samples were centrifuged (5000×*g*, 10 min, 4 °C), were washed with phosphate-buffered saline (PBS, 0.1 M, pH 7.4) and fixed with 2.5% (v/v) glutaraldehyde in 0.1 M sodium cacodylate trihydrate buffer for 2 h at 4 °C. Then, cells were fixed with 1% (w/v) osmium tetroxide (in 0.1 M cacodylate buffer) for 4 h at room temperature. Rewashed cells were dehydrated with acetone (50%, 70%, 95%, and 100%), followed by ethanol (30%, 50%, 70%, and 90%). Then 100% Epon Araldite resin was added and hardened for 48 h in a 60 °C oven. Thin sections were cut using a microtome (Reichert-Jung Ultracut E Ultramicrotome, EquipNet Inc., Canton, MA, USA) with a diamond knife (approximately 100 nm thin) and were placed on 300 mesh copper grids, which were then stained as follows: 2% aqueous uranyl acetate for 10 min, distilled water rinse for 2 × 5 min, lead citrate for 4 min, a quick rinse with distilled water and air dry. Samples were observed using a transmission electron microscope (JEM 1230, JEOL Inc., Peabody, MA, USA) at 80 kV, and images were captured using a digital camera (ORCA-HR, Hamamatsu Photonics, Japan). An average of 200 cells from two independent experiments was analyzed per each treatment. Morphological changes were quantified as a percentage of ruptured and dead cells.

### Cytoplasmic content leakage

#### Release of 260–280-nm absorbing materials

The integrity of the cell membrane of carvacrol-treated cells was evaluated by measuring the release of cell constituents at 260 nm and 280 nm. Briefly, 10 mL of cell cultures were incubated at 37 °C under agitation for 24 h in the presence of carvacrol (1/8 × MIC to 4 × MIC) and without carvacrol as control. Samples were centrifuged (5000×*g*, 10 min), and the absorbance of the supernatants was measured at 260 nm and 280 nm using NanoQuant Plate (Tecan Infinite™ M200 PRO, Morrisville, NC, USA) and were analyzed by agarose gel electrophoresis.

#### Release of cytoplasmic nucleic acids

Bacteria from the logarithmic phase of growth were collected and centrifuged (10,000×*g*, 10 min), washed once with 10 mM. PBS (pH 7.2) and resuspended to an OD_600_ = 0.6. 1 mL suspensions were treated with carvacrol (1/8 × MIC, 1/4 × MIC, 1/2 × MIC, MIC, 2 × MIC, and 4 × MIC) at 37 °C for 2 h and centrifuged (10,000×*g*, 5 min). The supernatants were analyzed by agarose gel electrophoresis. Ethanol precipitations were performed to isolate released nucleic acids, and precipitated pellets were dissolved with 10 µL of Tris–EDTA (TE) buffer (10 mM Tris–HCl, pH 7.5). The 10 μL of each sample was then mixed with 2 μL of 6× gel loading dye (B7024S, New England Biolabs, Ipswich, MA, USA). Carvacrol triggered nucleic acid leakage was visualized using a Bio-Rad ChemiDoc XRS + system following electrophoresis in 0.8% agarose gel containing ethidium bromide, with 1× TAE buffer [40 mM Tris base, 0.5 mM EDTA (pH 8.0) and 20 mM glacial acetic acid] at 120–140 V for 40–45 min. A 100 bp DNA ladder (UBPBio, Lucerna-Chem AG, CH, Luzern, Switzerland) was used as a molecular-weight size marker.

### Cell cytotoxicity assay

Cell cytotoxicity was determined using tonsil epithelial cells (HTonEpiCs) (ScienCell Research Laboratory, San Diego, CA, USA)^59^. Briefly, TonEpiC cells (6000 cells/100 μL) were seeded in poly-L-lysine-coated 96-well plates, and media was discarded after 24 h-incubation (5% CO_2_ at 37 °C) without disturbing to the adhered cell layer. A 100 μL of fresh media containing carvacrol was added. A mixture of LTA and PGN (5 mg/mL from each) was used as bacterial antigen controls. After 24 h-incubation, 10 μL of MTS: PMS (20:1) was added. The absorbance was measured at 490 nm after a 2.5 h-incubation, and results were expressed as percent cell viability compared with untreated cells.

### Release of cytoplasmic LDH for bacteria and tonsil cells

The LDH activity in the cell supernatant was measured using the CytoTox 96 Non-Radioactive Cytotoxicity kit (Promega Corporation, Fitchburg, WI, USA) according to the manufacturer’s protocol. The 24-h seeded cells (6000 cells/ 100 µL density) were washed and replaced with 100 µL of fresh growth medium. Both cells and bacterial suspensions were treated with DMSO or carvacrol (1/2 × MIC, MIC, 2 × MIC) at 37 °C. After 4 h incubation, cells/bacteria were centrifuged, and 100 µL of supernatant was mixed with 100 µL of CytoTox96 reagent. After 30 min at room temperature, acetic acid (1 M) was added to stop the reaction, and absorbance was measured at 490 nm. Released LDH was calculated by comparing cellular/bacterial LDH obtained by carvacrol treated supernatant with lysis buffer (9% Triton X-100).

### Statistical analysis

Statistical analyses were performed using MINITAB statistical software (Version 17.0; Inc., Chicago, IL, USA) and GraphPad Software 5.0 (La Jolla, San Diego, CA, USA). All the experiments were performed three times with triplicates. The data were presented as means ± standard errors. The mean separations were analyzed using Student’s t-test and one-way analysis of variance using Tukey’s test; Differences were considered statistically significant at *P < 0.05, **P < 0.01, and ***P < 0.001.

## Supplementary Information

Supplementary Information.
