# Supplementary material for: Carvacrol exhibits rapid bactericidal activity against Streptococcus pyogenes through cell membrane damage
Source: Sci Rep. 2021 Jan 15;11:1487. doi: 10.1038/s41598-020-79713-0 (PMC7811018; doi:10.1038/s41598-020-79713-0)
Supplement: Supplementary file 1 — Supplementary Information. [file 41598_2020_79713_MOESM1_ESM.docx]

**Supplementary material**

**Carvacrol exhibits rapid bactericidal activity against *Streptococcus pyogenes* through cell membrane damage**

Niluni M. Wijesundara, Song F. Lee, Zhenyu Cheng, Ross Davidson and H.P. Vasantha Rupasinghe


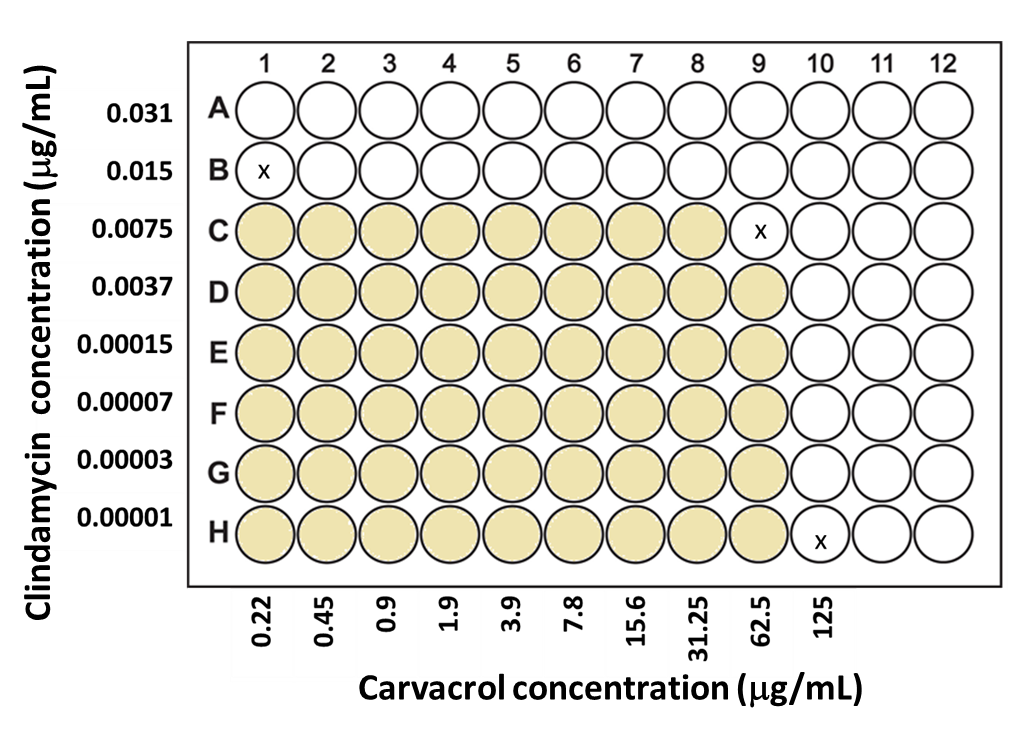


**Figure S1: Microdilution chequerboard method for fractional inhibitory concentration index (FICI) determination.** Shading wells represent the visible growth of *S. pyogenes.*
